# Extracellular volume by CMR is associated with serum biomarkers of extracellular matrix turnover and inflammation in hypertensive heart disease

**DOI:** 10.1186/1532-429X-18-S1-O103

**Published:** 2016-01-27

**Authors:** Peter W Shaw, Yang Yang, Jorge A Gonzalez, Sujith Kuruvilla, Matthew Gottbrecht, Fred Epstein, Li-ming Gan, Ellen C Keeley, Christopher M Kramer, Michael Salerno

**Affiliations:** 1 grid.27755.32000000009136933XCardiology, University of Virginia, Charlottesville, VA USA; 2 grid.27755.32000000009136933XBiomedical Engineering, University of Virginia, Charlottesville, VA USA; 3 grid.27755.32000000009136933XInternal Medicine, University of Virginia, Charlottesville, VA USA; 4 CVMD Innovative Medicine Unit, AstraZeneca R&D, Molndal, Sweden; 5 grid.27755.32000000009136933XRadiology, University of Virginia, Charlottesville, VA USA

## Background

Cardiac biomarkers have become increasingly important in cardiovascular disease. With the ability to detect focal and diffuse myocardial fibrosis, CMR is an important imaging biomarker. Diffuse myocardial fibrosis, reflected by increased extracellular volume (ECV), may underlie increased cardiovascular risk. ECV could provide a novel tool to evaluate the association myocardial-specific fibrosis and serum biomarkers. We hypothesized that ECV would correlate with known cardiovascular serum biomarkers in hypertensive heart disease.

## Methods

Patients with a history of hypertension with or without LVH by any non-invasive imaging modality and healthy volunteers (18 HTN, 13 HTN-LVH, and 13 controls) underwent CMR on a Siemens 1.5T Avanto and blood samples were obtained for biomarker analysis. T1 mapping was performed, using a previously validated modified Look-Locker inversion-recovery (MOLLI) pulse sequence, before 0.15 mmol/kg gadolinium DTPA (native T1) and at 10, 15, and 20 min post-contrast. Mean ECV and native T1 values were determined. Biomarkers were analyzed using the Proseek Multiplex CVD 96 × 96 (Olink Bioscience, Uppsala, Sweden). CMR parameters were compared with blood biomarkers via Pearson's correlation.

## Results

**Figure 1:**
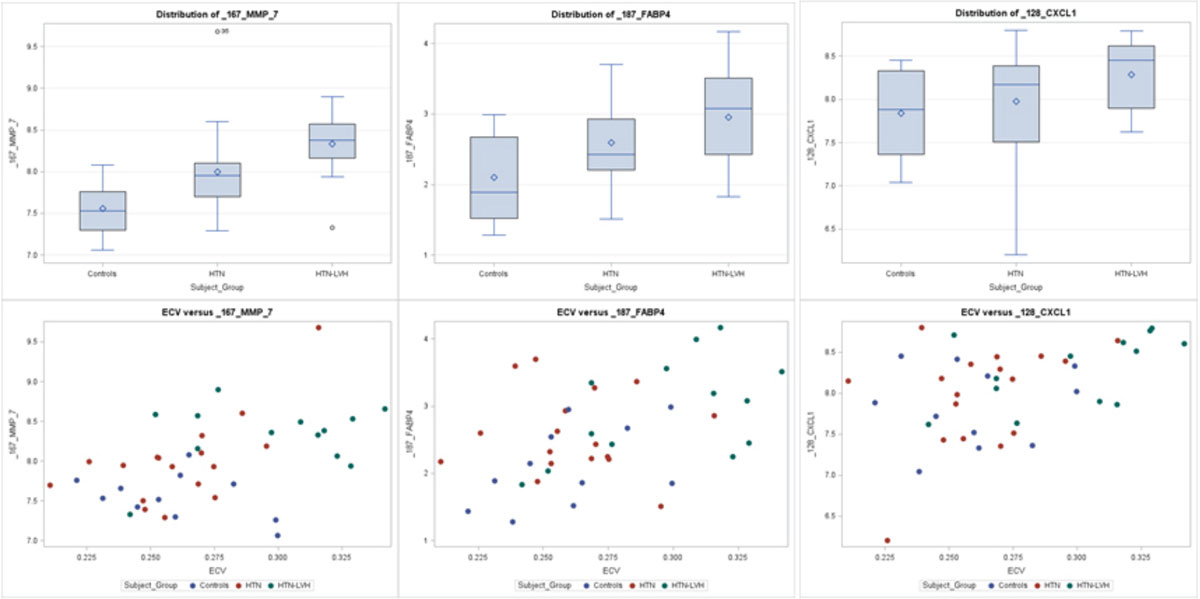
**1st row: Box Plot of MMP-7, FABP4, and CXCL1 levels separated by group; 2nd row: Correlation of ECV and cardiac biomarkers**.

Mean age was 57 ± 7, 64 ± 13, and 57 ± 12 for control, HTN, and HTN-LVH groups, respectively. LV mass index (LVMI) (g/m^2^) was 49.0 ± 6.8, 54.7 ± 10.6, and 82.8 ± 14.1* and ECV was 0.25 ± 0.02, 0.26 ± 0.03, 0.30 ± 0.03* (*p < 0.05 vs. controls and HTN) respectively. Matrix metalloproteinase 7 (MMP-7), C-X-C motif chemokine 1 (CXCL1), and fatty acid binding protein 4 (FABP4) had the highest correlation with ECV in all groups (r values: 0.46; 0.45; 0.44; respectively, p < 0.005 for all) (Fig 1 [CK1]). LVMI was highly correlated with T cell immunoglobulin and mucin domain (TIM-1) and MMP-7 as well (r values: 0.55; 0.46; respectively, p < 0.005 for all).

## Conclusions

Increased ECV, seen in patients with HTN-LVH, corresponds with diffuse fibrosis and may be due in large part to systemic inflammation and resulting alterations and remodeling of the extracellular matrix. MMP-7 is involved in extracellular matrix remodeling and is known to be upregulated in cardiac fibrosis and LVH. CXCL-1 and FABP4 also play a role in inflammation. Recent evidence suggests that FABP4 is involved in the integration of metabolic and inflammatory pathways and may play important roles in insulin resistance, atherosclerosis and diastolic dysfunction. The novel finding of the correlation with ECV with these markers suggests a link between altered metabolism and inflammation with cardiac fibrosis.

